# Impacts of elevated CO_2_ on exogenous *Bacillus thuringiensis* toxins and transgene expression in transgenic rice under different levels of nitrogen

**DOI:** 10.1038/s41598-017-15321-9

**Published:** 2017-11-07

**Authors:** Shoulin Jiang, Yongqing Lu, Yang Dai, Lei Qian, Adnan Bodlah Muhammad, Teng Li, Guijun Wan, Megha N. Parajulee, Fajun Chen

**Affiliations:** 1 Department of Entomology, Nanjing Agricultural University, Nanjing, 210095 China; 2 Texas A&M University AgriLife Research and Extension Center, Lubbock, TX USA

## Abstract

Recent studies have highlighted great challenges of transgene silencing for transgenic plants facing climate change. In order to understand the impacts of elevated CO_2_ on exogenous *Bacillus thuringiensis* (*Bt*) toxins and transgene expression in transgenic rice under different levels of N-fertilizer supply, we investigated the biomass, exogenous *Bt* toxins, *Bt*-transgene expression and methylation status in *Bt* rice exposed to two levels of CO_2_ concentrations and nitrogen (N) supply (1/8, 1/4, 1/2, 1 and 2 N). It is elucidated that the increased levels of global atmospheric CO_2_ concentration will trigger up-regulation of *Bt* toxin expression in transgenic rice, especially with appropriate increase of N fertilizer supply, while, to some extent, the exogenous *Bt*-transgene expression is reduced at sub-N levels (1/4 and 1/2N), even though the total protein of plant tissues is reduced and the plant growth is restricted. The unpredictable and stochastic occurrence of transgene silencing and epigenetic alternations remains unresolved for most transgenic plants. It is expected that N fertilization supply may promote the expression of transgenic Bt toxin in transgenic Bt rice, particularly under elevated CO_2_.

## Introduction

Global atmospheric CO_2_ concentrations have been increasing at an accelerating rate from 280 ppm before industrialization to 402 ppm in recent years (Mauna Loa Observatory: NOAA-ESRL), and are anticipated to reach at least 550 ppm by the year 2050^1^. Also, CO_2_ has been attracting people’s attention owing to its “greenhouse effects”, which is closely related to more frequent extreme weather events^2^. Typically, the effects of elevated CO_2_ on plants are generally characterized by increases in the photosynthetic rate, biomass, and carbon (C): nitrogen (N) ratio, especially in C_3_ crops^3–5^. Elevated CO_2_ can alter plant phenotype and chemistry by inducing changes in assimilation and re-assignment of C and N resources to primary and secondary metabolites in plant tissues, which, in turn, affects life-history and feeding responses of herbivorous insects^6–10^.

*Bacillus thuringiensis* (*Bt*) is a ubiquitous gram-positive and spore-forming bacterium, which produces specific insecticidal crystal proteins known as δ-endotoxins^11,12^. Based on amino acid sequence similarity and protein function, *Bt* δ-endotoxins are classified into two major groups, that is, *Cry* and *Crt* proteins^13^. The *Cry* toxins have been shown toxic towards a wide variety of larval stages of the Lepidoptera, Diptera, Coleoptera, Hymenoptera, Homoptera, Orthoptera, Mallophaga, Nematoda and Acarina^14,15^. *Bt* toxins are proven alternatives or supplement to synthetic chemical pesticides in commercial agriculture, forest management, and public health. However, directly transferring wild-type *Bt* genes into plants resulted in poor expression and potency, with the toxin comprising less than 0.005% of the total proteins in the plant, making transgenic plants still susceptible to insects even if the transgenes were driven by a strong plant promoter^16–18^. Thus, the usage of synthetic genes to express hybrid *Bt* toxins is regarded as a promising strategy in genetic engineering. A chimeric *Cry1Ab*/*Ac* gene, a fusion of *Cry1Ab* (GenBank Accession No. ×54939) and *Cry1Ac* (GenBank Accession No. Y09787) into a single gene, is highly toxic to three important rice lepidopteran pests, including *Chilo suppressalis*, *Scirpophaga incertulas*, and *Cnaphalocrocis medinalis* ^19,20^. Because of the excellent efficiency against lepidopteran pests and its environmental friendliness, China’s Ministry of Agriculture granted safety certificates in 2009 to two transgenic varieties of rice with hybrid *Bt* toxins for commercialization: a restorer line (cv. *Bt* Huahui-1) and a hybrid line (cv. *Bt* Shanyou-63), both of which expressed fused *Cry1Ab*/*Cry1Ac* genes^20^.

It is speculated that global agriculture will face numerous challenges owing to climate change^1^. Elevated CO_2_ can have various effects on different trophic levels, plants, herbivores, and predators/parasitoids^3,6,7,9,10,21^. Under these circumstances, transgenic plants may become a significantly greater component of cropping systems for sustainable agriculture. However, the performance of transgenic plants, the stability of the transgenic traits, and their ecological interactions have been studied under atmospheres with elevated CO_2_^22–25^. Several studies suggested that the expression of exogenous gene in transgenic plants diverts some nutrients from the normal physiological pathways which could alter the C–N balance, especially under changed abiotic conditions^23–26^. Coviella and Trumble^22^ and Tsutsumi *et al*.^27^ reported that applied N fertilization can relieve such nutrient diversion. N fertilization is, thus, regarded as an attractive strategy to improve the C-N balance in transgenic plants under elevated CO_2_ in the future^22,23^.

Methylation of cytosines in DNA is a common eukaryotic DNA modification that functions as a powerful mechanism to regulate gene expression through gene silencing^28–30^. Napoli *et al*.^31^ and Van der Krol *et al*.^32^ introduced *CHS* (chalcone synthase) gene and *DFR* (dihydroflavonol-4-reductase) gene into petunia which was expected to overexpress the floral pigmentation. Unexpectedly, the introduced transgene created a blockage in anthocyanin biosynthesis. Napoli *et al*.^31^ and Reddy *et al*.^33^ demonstrated that the transgene silencing occurred in transgenic plant, and suggested that it might be connected to methylation. Subsequent studies found much more transgene silencing in transgenic plants^34,35^ as well as in fungi^36^ and higher order animal taxa^37,38^. Numerous researches have indicated that transgenes are susceptible to silencing in all studied plant species^39,40^, including rice^41^. Silencing resulting from interactions among multiple copies of transgenes or when additional copies of an endogenous gene are expressed ectopically involves homology dependent gene silencing (HDGS). The mechanism of HDGS is not yet clearly understood, though a number of speculative hypotheses have been put forward and extensively reviewed^42–44^. Basically two forms of transgene silencing have been described, transcriptional gene silencing (TGS), in which gene expression is directly blocked, and posttranscriptional gene silencing (PTGS) in which mRNA is degraded^45^. Both types of silencing are associated with de novo methylation of cognate sequences: in TGS, DNA methylation occurs in the promoter region^46,47^, and in PTGS, it is associated with DNA methylation in the coding sequences^48–50^.

In this study, we simulated the future global atmospheric CO_2_ concentrations to determine how elevated CO_2_ affects the *Bt*-transgene methylation and *Bt* toxins expression in transgenic *Bt* rice under different rates of N-fertilizer augmentation. Two hypotheses were issued: (1) The expression of *Bt* transgene should be down-regulation by hyper-methylation of transgenic rice facing elevated CO_2_ levels, and (2) N fertilization should relieve or remove the N limitation for transgenic *Bt* rice under elevated CO_2_.

## Materials and Methods

### Plant materials and growth conditions

**Figure 1:**
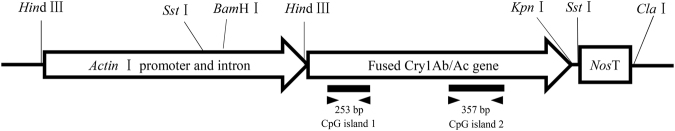
Schematic diagram of the fused *Cry1Ab/Ac* gene and its plasmid.

The generalized modified rice used in this experiment was the transgenic restorer line, Huahui-1, which was developed by using Minghui 63 as recipient to harbor the fusion gene *Cry 1Ab*/*Cry1Ac* from transgenic event TT51-1 (GenBank Accession Number: EU880444.1), provided by Huazhong Agricultural University (Wuhan, China). The transgene is regulated by the rice *actin 1* promoter and the *nopaline synthase* (*NOS*) gene terminator (Fig. 1).

Experiments were conducted at Nanjing Agricultural University in two electronically controlled growth incubators (GDN-400D-4/ CO_2_; Ningbo Southeast Instrument CO., LTD, Ningbo, China) with a gas-tank system that maintained the desired CO_2_ concentration. The CO_2_ concentrations in these two growth incubators were set at the current atmospheric CO_2_ level (400 ± 20 ppm) and at an elevated level (800 ± 50 ppm, slightly higher than the predicted level at the end of this century)^1^. Within each CO_2_ level, N-fertilizer sub-plot treatment was set at five levels, including 1/8, 1/4, 1/2, 1, and 2 N; the standard N-fertilizer supply or 1 N was 1.25 mM NH_4_NO_3_), delivered through NH_4_NO_3_. The entire experiment, thus, consisted of 2 CO_2_ concentrations ×5 N-fertilizer rates (total 10 treatment combinations). The two growth incubators were alternated by switching CO_2_ concentration rates as well as swapping the entire content of each chamber every five days in order to equalize the possible bias in plant growth due to incubator-specific growth conditions.

Seed germination was induced on a moist filter paper for 48 h and then the seeds were sown into plastic foam (0.7 cm in thickness) covering the plastic cups (9 cm in diameter and 6.5 cm in height). There were five holes in each piece of plastic foam and two seeds were transferred into each hole (2 rice plants per hole ×5 holes per piece of plastic foam ×1 piece of plastic foam per plastic cup ×4 plastic cups = 40 rice plants per treatment). The plastic cups were filled with modified culture solutions^51^, and the nutrient solution was renewed daily. The composition of modified culture solutions was as follows (per liter): (1) Macronutrient solution: NH_4_NO_3_, 1.25 mM; KH_2_PO_4_, 0.3 mM; K_2_SO_4_, 1 mM; CaCl_2_·2H_2_O, 1 mM; MgSO_4_·7H_2_O, 1 mM; Na_2_SiO_3_·9H_2_O, 0.5 mM. (2) Micronutrient solution: MnCl_2_·4H_2_O, 9 μM; Na_2_MoO_4_·2H_2_O, 0.39 μM; H_3_BO_3_, 20 μM; ZnSO_4_·7H_2_O, 0.77 μM; CuSO_4_·5H_2_O, 0.32 μM; FeSO_4_·7H_2_O + Na_2_-EDTA, 20 μM. All rice plants were grown at 26.5 ± 1.0 °C with 70 ± 10% humidity at a photoperiod of 14 h: 10 h (light/dark).

### The measurement of transgenic Bt rice biomass

At 35 days after planting (DAP), twenty rice seedlings were randomly selected to determine the biomass of aboveground (shoot) and belowground (root) plant tissues. Fresh weights of each of these two plant parts were recorded by using precision scales with an accuracy of ± 0.1 mg (Mettler Toledo AL104). These post-fresh weight samples were collected in the self-sealing bags separately and stored at −80 °C in an ultra-cold storage freezer (Thermo Scientific Forma 702, USA) for the bioassay of relative expression and methylation status of *Bt* transgene.

### The measurement of foliar Bt toxins and total soluble proteins of transgenic Bt rice

On 35 DAP, ten seedlings were randomly selected from the remaining twenty plants in each treatment combination for the measurement of foliar *Bt* toxins and total soluble proteins. Three leaves from each seedling were separately collected (approximately 30–40 mg total fresh weight), and were then placed into 1.5 ml microtube. All samples were homogenized in a TissueLyser II (Qiagen) by shaking for 2 min at 28 Hz with one steel ball per tube. After homogenization, the extraction buffer PBST was added into the tube at a ratio of 1:10 (tissue weight in g: buffer volume in ml) for *Bt* toxins test, and 0.9% saline was used as an extraction buffer at a ratio of 1:9 (tissue weight in g: buffer volume in ml) for total soluble proteins test, according to the specification respectively. The supernate of extraction buffer was used for the following test as protein solution. The foliar content of total soluble proteins was determined following the corresponding diagnostic kit A045-2 (Nanjing Jiancheng Bioengineering Institute), and the *Bt* toxins content was analyzed with an ELISA test (*Bt*-Cry1Ab/Ac ELISA Kit, Agdia, Elkhart, IN, USA) according to the manufacturer protocol and with Cry1Ab/Ac standards for quantitative determination. Each sample of the above two foliar content tests for *Bt* toxins and total soluble proteins was replicated three times.

### RNA preparation and reverse transcription

Total RNA was extracted from leaf tissues of the sampled seedlings by using TRIzol^®^ reagent (Invitrogen). The concentration and quality of samples was determined by NanoDrop^TM^ spectrophotometer (Thermo Scientific) and 1.5% agarose gel electrophoresis. The first-strand complementary cDNA templates were synthesized with 100 ng total RNA by using PrimeScript^TM^ RT reagent Kit with gDNA Eraser (TaKaRa, Japan). Reverse transcriptase reactions were performed in a 20 μl final volume reaction.

### Real-time PCR analysis

Each cDNA product was diluted from 20 µl to 200 µl with RNase-free dH_2_O, in order to make the Ct value within the suitable range of 15–35 based on preliminary experiments. For fluorescence-based quantitative real-time PCR (qRT-PCR), 2 µl cDNA dilution and 0.2 μM primers were used in 1 × SYBR^®^ *Premix Ex Taq* ^TM^ (TaKaRa, Japan) with 7500 Real-Time PCR Detection System (Applied Biosystems) following the supplier’s instructions. Reactions were performed in a 20 µl final volume. Specific primers for the fusion *Cry1Ab*/*Ac* gene were designed by Beacon Designer^TM^ 7.9 software, and the housekeeping genes *actin1* and *ubiquitin* were used as the internal standard to analyze target gene expression (Primers for qRT-PCR were as shown in Table 1). Quantification of the transcript level of target gene was conducted following the 2^−ΔΔCt^ normalization method^52^. The expression levels of internal control genes were examined in every PCR plate to eliminate systematic error. Three biological replicates were made for each treatment in the qRT-PCR analysis, and each biological replicate contained three technical repeats.

**Table 1:** Primers for qRT-PCR.

Primer	Sequence (5′-3′)	GeneBank Accession	Description
Cry1Ab/Ac-F	TAGAGTTCGTGTGAGGTA	EU816953	*Bt* protein gene
Cry1Ab/Ac-R	CTGTATTGGAGAAGATGGAT		
actin1-F	ATGGCAACATTGTGCTCAGTG	***Bt***130427^91^	Rice housekeeping gene
actin1-R	CCTCCGATCCAGACGCTGTA		
ubiquitin-F	GCTCCGTGGCGGTATCAT	NC_029258^92^	Rice housekeeping gene
ubiquitin-R	CGGCAGTTGACAGCCCTAG		

### Genomic DNA isolation of transgenic Bt rice leaves

Genomic DNA was isolated and purified from 40 mg leaf tissue samples prepared in *foliar Bt toxin and total protein estimation* section by using DNAsecure Plant Kit (TIANGEN, Beijing, China), following its manufacturer’s instructions. DNA was quantified by using NanoDrop^TM^ spectrophotometer (Thermo Scientific) and stored at −20 °C before use.

### Bisulfite genomic sequencing

DNA methylation analysis was performed by the bisulfite sequencing method. Because bisulfite treatment can convert unmethylated cytosines to uracils, it was used to determine the methylation status of cytosines in CG, CHG, and CHH sequences (where H could be A, T, or C). In order to obtain detailed information on the methylation status of cytosine which could represent the integral level of the transgene in silence performance, we used bisulfite sequencing, which allowed detection of mostly methylated cytosine residues within a DNA region of the *Bt*-transgene body. Two pairs of primers were designed to amplify methylation and non-methylation copies of the transgene, for acquiring major information on methylation of the transgene. Two PCR fragments (CpG island) were analyzed: P1 (CpG island 1) was amplified from the top strand of the transgene, and P2 (CpG island 2) was amplified from the bottom strand (Fig. 1).

Bisulfite treatment of genomic DNA was performed by using the DNA Bisulfite Conversion Kit (TIANGEN, Beijing, China). A total of 0.6 µg genomic DNA was converted for 70 min with the following program: 95 °C for 10 min, 64 °C for 60 min, and reactions were then maintained at 4 °C until the next step. The target sequences were amplified by PCR from the bisulfite-treated DNA (150 ng) by using the Methylation-specific Kit (TIANGEN, Beijing, China). The optimized PCR program consisted of 95 °C for 5 min, 35 cycles of 94 °C for 20 sec, 55 °C for 30 sec, 72 °C for 20 sec, and a final step with 72 °C for 5 min. The PCR products were excised and purified from the gel with AxyPrep DNA Gel Extraction Kit (Axygen, Union City, USA), and cloned into *pEASY* ^®^-T3 Cloning Vector using pEASY-T3 Cloning Kit (TransGen, Beijing, China) with *Trans* 1-T1 Phage Resistant Chemically Competent Cell (TransGen, Beijing, China). At least ten independent clones from each PCR product were sequenced (Table 2). Primers for bisulfite sequencing were designed using Methyl Primer Express Software^®^ (Applied Biosystems). The primer sequences and products are summarized in Fig. 1 and Table 3.

**Table 2:** The number of sequenced clones from each PCR product.

Number (clones)		Nitrogen concentration treatments				
		1/8 N	1/4 N	1/2 N	1 N	2 N
CO_2_ treatments	Ambient	15	14	19	16	14
	Elevated	14	13	10	12	10

**Table 3:** Primers for bisulfite sequencing.

Primer	Sequence (5′-3′)	GeneBank Accession	Description	Designed by
P1-F	TTGGTGTAAATTGAGTAGTTGAT	EU880444.1	CpG island 1	Methyl Primer Express Software v1.0
P1-R	ACACRAACAAAAAAAAAACTTA			
P2-F	GGAGAGTATTATTGGTTTGGATA	EU880444.1	CpG island 2	Methyl Primer Express Software v1.0
P2-R	CAACCTATAAAAAAATCCTTACCT			

### Data Analysis

Statistical analysis of data was performed by using SPSS 20.0 (SPSS Inc., Chicago IL, USA). The biomass of different rice plant parts, total soluble proteins and *Bt* toxins in foliar contents, and foliar *Bt-*transgene (i.e., *Cry1Ab*/*Ac*) expression levels were analyzed by a two-way analysis of variance (ANOVA) with CO_2_ concentration as main factor (ambient CO_2_ *vs*. elevated CO_2_) and N-fertilizer supply (5 levels: 1/8, 1/4, 1/2, 1 and 2 N) as sub-factor. If significant effects of CO_2_ concentration, N-fertilizer supply, and their interactions were found, the two-tailed Student’s t-test was used to compare the means between ambient CO_2_ and elevated CO_2_ at *P* < 0.05, and the least significant difference (LSD) test was used to separate the treatment means. Sequences were manually trimmed and the data analysis performed with the online tool CyMATE (http://cymate.org/).

## Results

### Aboveground and belowground biomass

**Figure 2:**
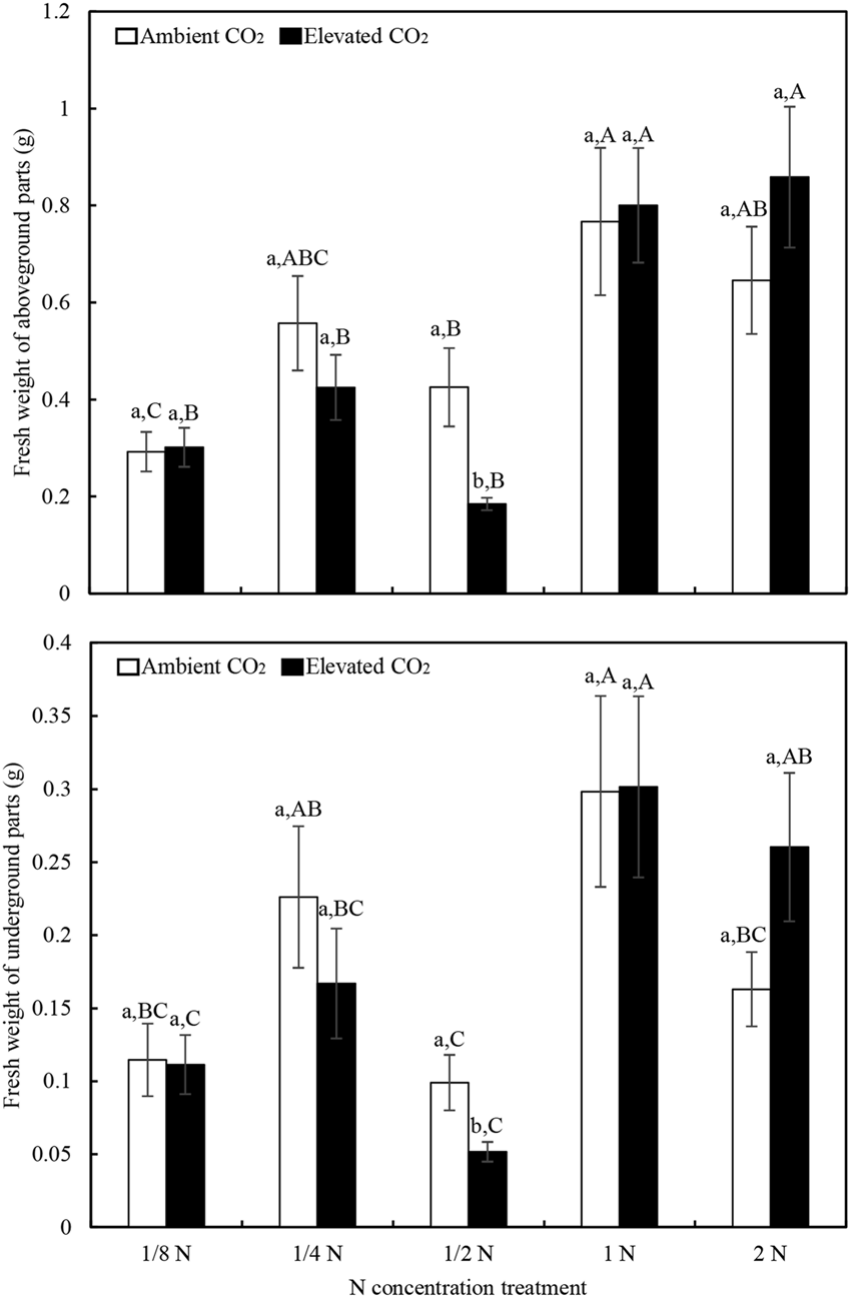
Aboveground (**A**) and belowground (**B**) biomass of transgenic *Bt* rice plants grown under ambient and elevated CO_2_ at different N-fertilizer augmentation rates. Different lowercase and uppercase letters indicate significant differences between the ambient CO_2_ and elevated CO_2_ within N-fertilizer rates, and between the different N-fertilizer rates within CO_2_ level by LSD test at *P* < 0.05.

The results showed that the aboveground and belowground biomasses of transgenic *Bt* rice were significantly affected by N supply rates (Table 4). Also, the augmentation of 1 N (1.25 mM NH_4_NO_3_) as the standard level of N fertilization was the optimal N condition for the biomass production regardless of the CO_2_ level. The aboveground and belowground biomass of transgenic *Bt* rice was increased by elevated CO_2_ in comparison with ambient CO_2_ at the 2 N level, but the effect of elevated CO_2_ on rice seedling biomass decreased at sub-optimal levels of N (i.e., 1/8, 1/4 and 1/2 N), with a conspicuous sharp decrease at 1/2 N (Fig. 2).

**Table 4:** F-values and P-values from two-way ANOVA for the effects of CO_2_ levels and N levels on the fresh weight of rice plants, total soluble protein, *Bt* toxin content, and *Bt* gene expression.

Parameters	df	CO_2_		N		CO_2_ × N	
		*F*-values	*P*-values	*F*-values	*P*-values	*F*-values	*P*-values
Fresh weight of aboveground parts (g)	199	0.148	0.701	11.677	0.000***	1.578	0.182
Fresh weight of belowground parts (g)	199	0.003	0.953	9.252	0.000***	1.422	0.228
Total soluble protein (mg/g leaf fresh weight)	99	0.661	0.419	26.577	0.000***	14.250	0.000***
*Bt* protein content (mg/g leaf fresh weight)	99	13.937	0.000***	11.820	0.000***	2.475	0.050*
*Bt* gene expression	29	0.002	0.969	26.130	0.000***	10.597	0.000***

*P < 0.05; ***P < 0.001.

### Foliar Bt toxins and total soluble proteins

**Figure 3:**
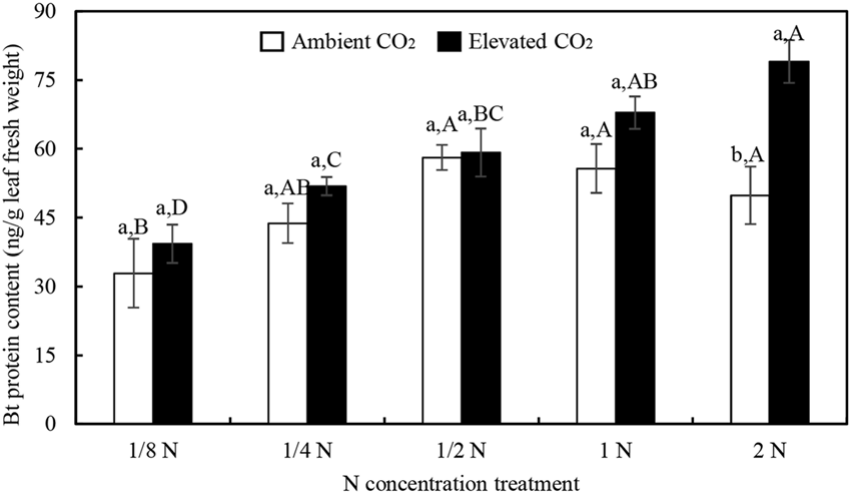
The foliar *Bt* protein contents of transgenic *Bt* rice grown under ambient and elevated CO_2_ with different N-fertilizer augmentation rates. Different lowercase and uppercase letters indicate significant differences between the ambient CO_2_ and elevated CO_2_ within N-fertilizer rate, and between the different N-fertilizer rates within CO_2_ level by LSD test at *P* < 0.05.

**Figure 4:**
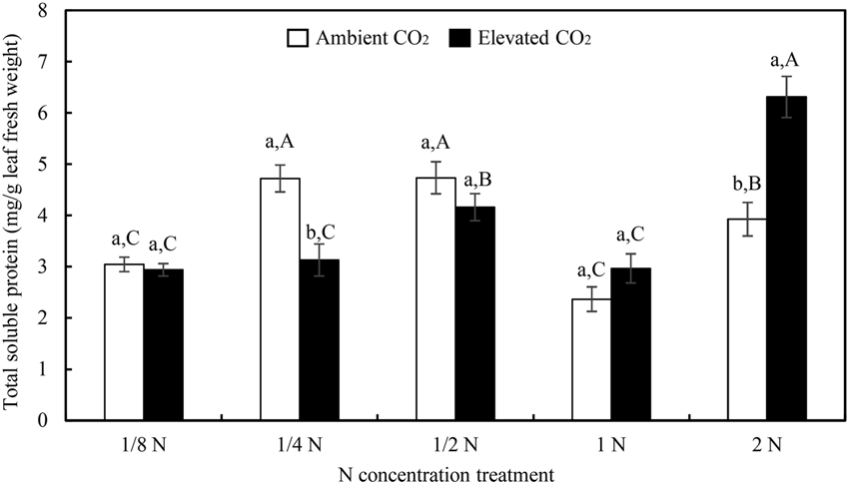
The foliar total soluble protein of transgenic *Bt* rice grown under ambient and elevated CO_2_ with different N-fertilizer augmentation rates. Different lowercase and uppercase letters indicate significant differences between the ambient CO_2_ and elevated CO_2_ within N-fertilizer rate, and between the different N-fertilizer rates within CO_2_ level by LSD test at *P* < 0.05.

CO_2_ level, N augmentation rates, and CO_2_ × N interactions all significantly influenced foliar *Bt* toxin content on transgenic rice while N augmentation rates and CO_2_ × N interactions significantly affected the total soluble proteins (Table 4). Transgenic *Bt* rice grown under ambient CO_2_ contained 32.89 μg *Bt* toxins/g foliar fresh weight at 1/8 N level and showed rising tendency until about 1/2 N level, but the further increase in N rates did not correspondingly increase the *Bt* toxin (Fig. 3). Compared with ambient CO_2_, elevated CO_2_ increased foliar *Bt* toxin content on transgenic *Bt* rice with increased N-fertilizer augmentation, and there was significant increase at 2 N level (Fig. 3). However, elevated CO_2_ treatment showed no significant influence on the foliar total soluble proteins at optimal (standard) and sub-optimal N levels, except at 1/4 N, but significantly increased total soluble proteins was observed at 2 N level (Fig. 4).

### Bt transgene expression on leaves

**Figure 5:**
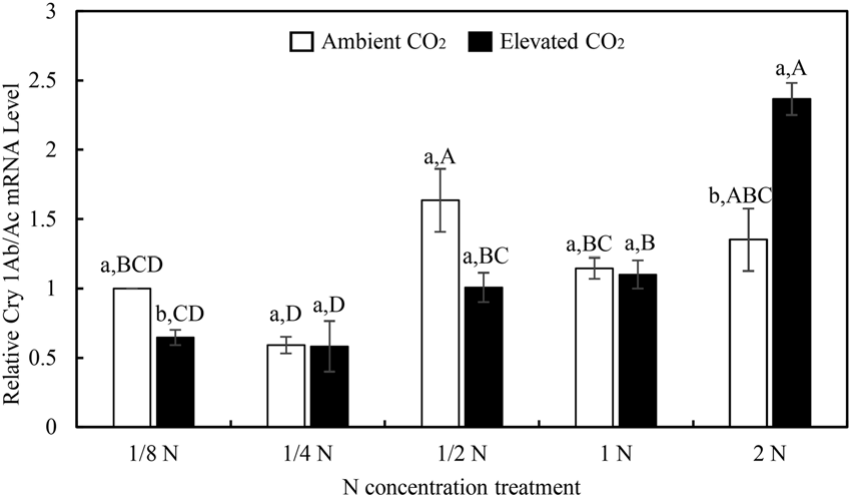
The relative transcript levels of fused *Cry 1Ab/Ac* gene in leaves of transgenic *Bt* rice grown under ambient and elevated CO_2_ with different N-fertilizer rates. Different lowercase and uppercase letters indicate significant differences between the ambient CO_2_ and elevated CO_2_ within N-fertilizer rate, and between the different N-fertilizer rates within CO_2_ level by LSD test at *P* < 0.05.

Two-way ANOVA indicated that N-fertilizer augmentation and CO_2_ × N interactions had significant effects on *Bt* transgene expression (Table 4). Under ambient CO_2_ growing conditions, the expression of *Bt trans*gene was up-regulated with increased N-fertilizer supply from 1/8 to 1/2 N, but the transgene expression stabilized beyond the 1/4 N and 1/2 N levels. However, increased N supply significantly up-regulated the *Bt*-transgene expression beyond optimum supply of nitrogen under elevated CO_2_, especially at 2 N (Fig. 5). Compared with ambient CO_2_, elevated CO_2_ significantly down-regulated the *Bt*-transgene expression (35.31%) at the lowest N-fertilizer rate (i.e., 1/8 N) while significantly up-regulated the *Bt*-transgene expression (75.13%) at the highest N-fertilizer rate (i.e., 2 N) (Fig. 5).

### Methylation status of Bt-transgene of transgenic Bt rice leaves

**Figure 6:**
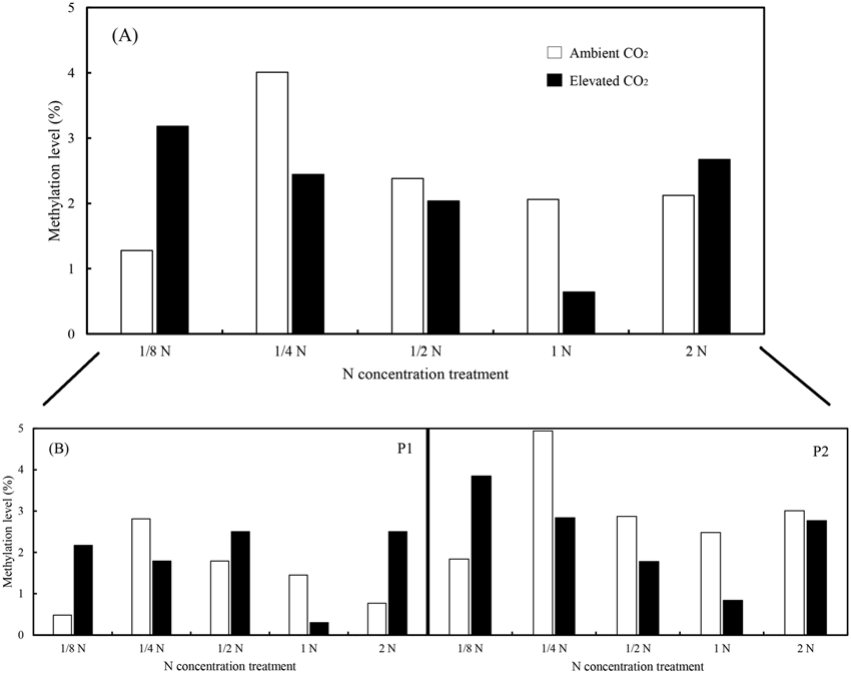
The cytosine methylation of fused *Cry 1Ab/Ac* transgene in leaves of transgenic *Bt* rice grown under ambient and elevated CO_2_ with different N-fertilizer rates. (n = minimum of 10 clones for each treatment; (**A**) Percentage methylation of the fused *Cry 1Ab/Ac* transgene (both two fragments); (**B**) Percentage methylation of P1 and P2 fragments in the fused *Cry 1Ab/Ac* transgene, respectively).

The methylation status of *Bt* transgene (i.e., *Cry1Ab*/*Ac* fusion gene) transferred to rice plant showed uneven methylation under different combination treatments of CO_2_ concentration and N-fertilizer supply (Fig. 6A). Different N supply showed various effects towards the methylation of *Bt*-transgene body. Under ambient CO_2_, lowest and highest methylation percentages were observed at 1/8 N (1.28% of cytosines) and 1/4 N (4.01% of cytosines), respectively; methylation was stabilized at ½ N to 2 N range (2.06% at 1 N level to 2.38% at ½ N level of cytosines). In contrast, under elevated CO_2_, the methylation status showed declining tendency at sub-optimal levels of N (3.18% to 0.64% of cytosines), and then it moved to a hypermethylation status at 2 N level (2.67% of cytosines) (Fig. 6A). Furthermore, at severe nitrogen-deficit (1/8 N) and excessive nitrogen fertility (2 N) conditions, *Bt*-transgene body appeared to undergo hypermethylation under elevated CO_2_ (3.18% and 2.67% of cytosines) compared to ambient CO_2_ (1.28% and 2.12% of cytosines), respectively. In contrast, elevated CO_2_ (2.44%, 2.04%, and 0.64% of cytosines) led lower methylation in *Bt*-transgene body than ambient CO_2_ (4.01%, 2.38%, and 2.06% of cytosines) at intermediate N levels (i.e., 1/4, 1/2, 1 N) (Fig. 6A).

**Figure 7:**
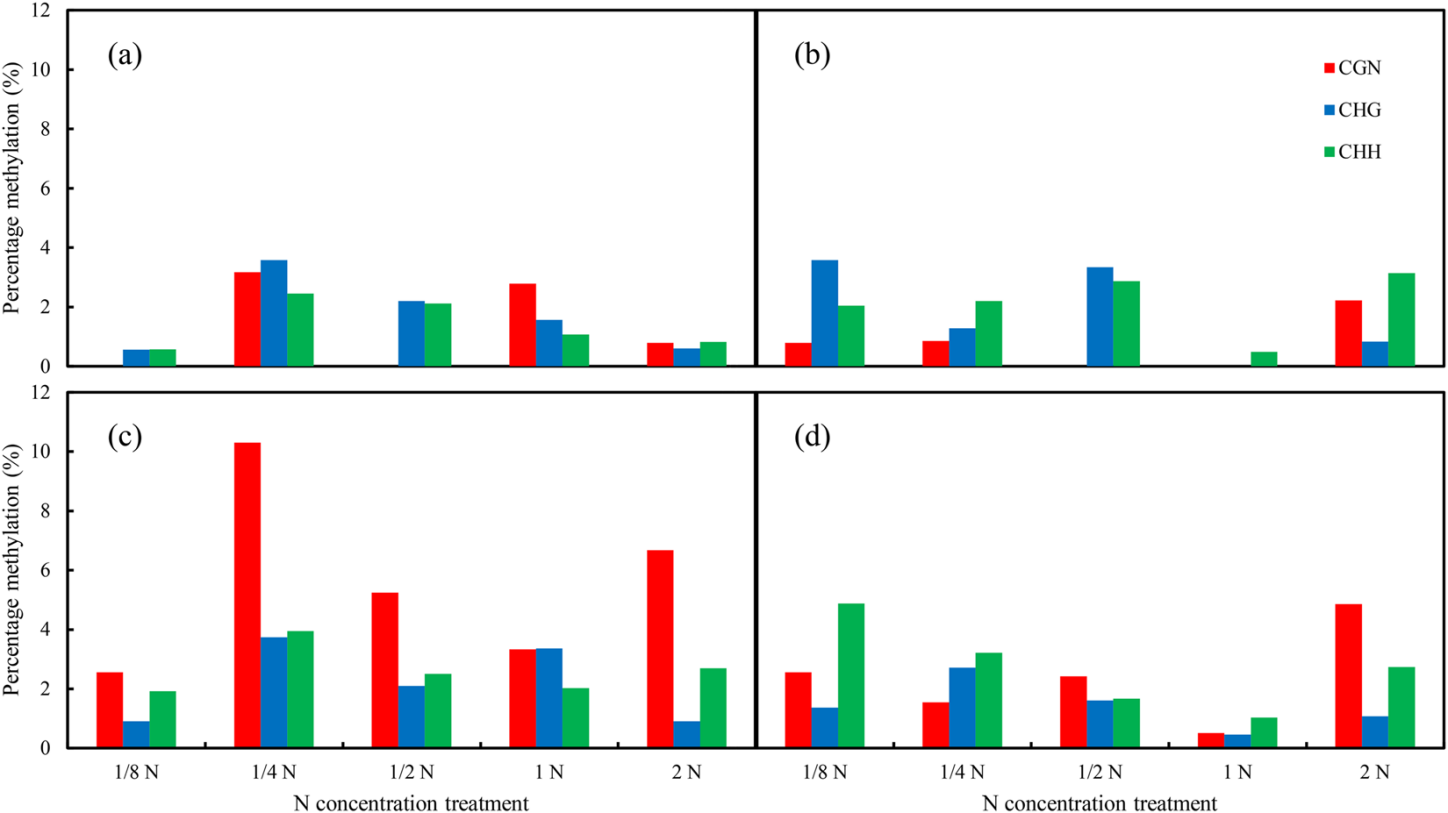
Percentage methylation of different methylation patterns (CGN, CHG and CHH) in P1 (**A**,**B**) and P2 (**C**,**D**) fragments of the fused *Cry 1Ab/Ac* transgene in leaves of transgenic *Bt* rice grown under ambient (**A**,**C**) and elevated (**B**,**D**) CO_2_ with different N-fertilizer rates.

The integral methylation level of fragment P1 was lower than P2 under same treatments, except for 1/2 N level under elevated CO_2_. At 1/8, 1/4 and 1 N levels, the methylation status of P1 or P2 manifested a similar tendency as the integral level of this *Bt*-transgene body under CO_2_ treatment (Fig. 6B). However, at 1/2 and 2 N levels, elevated CO_2_ increased methylation frequency in fragment P1, and it had inverse effects on methylation frequency in fragment P2 (Fig. 6B). Moreover, within the 253 bp fragment P1, a total of 9 CG, 12 CHG, and 35 CHH sites were found as potential targets for methylation, while in 357 bp fragment P2, there were 15 CG, 17 CHG and 60 CHH sites as potential targets for methylation. Also, the methylation of cytosines located at CHG and CHH sites appeared to be higher in fragment P1 (Fig. 7, Supplementary Figs 1 and 2). Conversely, the CG site preference was more apparent in fragment P2 compared with a low level of methylation in fragment P1 (Fig. 7, Supplementary Figs 3 and 4).

## Discussion

Global climate change has introduced a new challenge to the current plant genetic improvement programs^53,54^. Previous studies focused on plants grown under elevated CO_2_ have suggested that there is a link between ‘up-regulation’ and ‘down-regulation’ of plant growth traits^55^. Moutinho-Pereira *et al*.^56^ reported increased leaf tissue thickness in grape plants (*Vitis vinifera* L.) as a growth response to elevated CO_2_. Guo *et al*.^57^ and Reich *et al*.^58^ noted that the elevated CO_2_ resulted in increased plant biomass in *Medicago truncatula* (63.3%) and grassland (33%), respectively. While the occurrence of down-regulation of photosynthesis has been shown in several free-air CO_2_ enrichment (FACE) experiments, it has not been a consistent phenomenon^55^. A short-term effect of elevated CO_2_ may be misleading when attempting to predict longer-term effects^59^. Moreover, the effect of elevated CO_2_ on plant biomass of transgenic plants is quite complex, e.g., increased leaf biomass, increased leaf area and plant height^26,60^, in contrast, there was different conjecture based on their results, because heterologous protein produced by transgenic plant, as a kind of toxic molecule, could impair the plant tissues, which in turn progressively damaged host cells^61^. In this study, we first investigated the interactive effect of various levels of N fertilizer augmentation and elevated CO_2_ on transgenic rice biomass. The results suggested that there is a positive effect of elevated CO_2_ on biomass at increased N augmentation (>1 N level), but on the contrary, elevated CO_2_ resulted in decreased plant biomass under N-deficit production regimes (1/2 and 1/4 N levels). Based on these results, we conclude that elevated CO2 and N-fertilizer supply both make an effect on foliar biomass. It appears that the increased CO_2_ puts a higher N demand on transgenic rice to achieve the same level of biomass.

At high N levels, CO_2_ concentration may prove to be a limiting factor affecting plant photosynthesis. Yamori *et al*.^62^ and Adachi *et al*.^63^ reported that the photosynthesis of rice leaves is limited by CO_2_ concentration, which showed positive correlation, at carboxylation sites inside chloroplast when nitrogen fertilizer is controlled. During photosynthesis, CO_2_ molecules in the air diffuse through the stomata into the substomatal cavities and then move via cell walls, plasmalemma, cytosol, and chloroplast envelope membranes to the carboxylation sites in the stroma^64^. While there is a different response pattern at low N levels, N supply may play a more important factor than CO_2_ concentration towards plant growth. The effects of CO_2_ concentration may be weaker when plant growth is limited by nutrient availability^58^. Several studies have shown that N promotes photosynthesis which has a strong and positive correlation with biomass accumulation by increasing Rubisco content and CO_2_ diffusion conductance in rice leaves^62,65,66^. Sub-optimal levels of N may lead to reduction in total fresh, as well as dry weight of leaf blades, leaf sheaths plus stems, and total shoot weight in rice, but the cultivar response to N supply varies^67^. The N limitation feedback hypothesis suggests that the negative impacts of elevated CO_2_ on N cycling can constrain responses of plants to elevated CO_2_ and it may also be induced by the low supply of N^67–69^. Nguyen *et al*.^67^ reported that nitrogen use efficiency (NUE) increased gradually when lowering the N supply from 1 N to 1/8 N, while the NUE was maximized at the 1/2 or 1/4 N levels. Elevated CO_2_ appeared to have varying effects on the N concentrations in different plant types, with greater reduction of N concentration in C_3_ plants than in C_4_ plants. Cotrufo *et al*.^70^ reported that plants exposed to elevated CO_2_ altered their N allocation between above- and below-ground components: root N concentrations were reduced by ~9% compared to ~14% reduction for above-ground tissues. Results of this study are in agreement with prior studies related to theory of multiple-resource-limitation^69–72^ as we demonstrated the separate effect of CO_2_ and N, as limiting factors, on plant biomass accumulation in transgenic rice.

Our results indicated that *Bt* protein content of rice plants under elevated CO_2_ was higher than that under ambient CO_2_ regardless of N treatment. In plants, low concentrations of reactive oxygen species (ROS) functions in signal transduction leading to activation of defense responses^73^, while higher levels of ROS lead to oxidative damage of lipids, DNA, and proteins^74^. Two plausible pathways exist for elevated CO_2_ to decrease oxidative stress via reduction in cellular production of ROS. First, increased CO_2_:O_2_ ratios within chloroplasts would decrease electron leakage from PSI to O_2_, thereby attenuating ·O_2_^−^ formation, while decreased photorespiration would reduce cellular H_2_O_2_ production associated with glycolate metabolism^75^. Second, growth at elevated CO_2_ often improves plant water status, which would indirectly decrease antioxidant activities that are stimulated by water stress^76^. So, the decreased oxidative stress may display less oxidative damage to proteins including *Bt* protein, as observed in our study. Leaf age may have many problematic effects to plant growth^77^. Leaf-specific differences were observed in chlorophyll and protein content and starch and metabolite accumulation, and elevated CO_2_ caused both suppression and promotion of CO_2_ assimilation within the same plant depending on leaf age^78^. Thus, the effects of elevated CO_2_ strongly depend on the developmental state of the leaves. As mentioned, both the aging of leaves and the adaptation towards elevated CO_2_ contributed to the changes in profiles of chlorophyll, protein, and several plant metabolites^79^, which might contribute to the results in our study.

In the current study, expression levels and methylation status of *Cry1 Ab*/*Ac* fusion gene were determined to estimate the potential mechanism of *Bt* toxins in response to elevated CO_2_ and N fertilization treatments. The expression of *Bt* transgene was up-regulated when rice plants were exposed to excessively sub-optimal N fertility (<1/2 N) and ambient CO_2_, whereas elevated CO_2_ significantly up-regulated the *Bt*-transgene expression only under an excessively high N regime (i.e., 2 N). Previous studies have noted that the expression of protein is controlled by not only mRNA through transcriptional level but also RNA-binding proteins and microRNAs through post-transcriptional level^80,81^, likewise, the expression of exogenous *Bt* gene was also regulated through this way. Post-transcriptional regulation is increasingly recognized as a complex system that controls every aspect of RNA metabolism, while it is mediated by the interactions of trans-factors such as RNA-binding proteins and microRNAs with cis-acting elements located in mRNAs. RNA-binding proteins critically regulate mRNA splicing, localization, degradation and translation by binding to short sequences and/or structure motifs in target mRNAs^81,82^. Likewise, microRNAs can be sequestered and neutralized by the target mRNAs in addition to competition between binding sites on different mRNAs, which shows the fundamental principle of post-transcriptional regulation^80,83^. We speculate that the difference in *Bt* expression at various N augmentation rates as modulated by CO_2_ level may be due to the variation in RNA-binding proteins. Jens and Rajewsky^80^ reported that mRNA un-translated regions flanked the coding sequence and were bound by post-transcriptional regulators (RNA-binding proteins and microRNAs), which collectively controlled mRNA stability, mRNA localization and protein production. Melanson *et al*.^84^ demonstrated that the synthetic novel cis-acting elements from 3′ un-translated region of DNA damage-binding protein 2 can lead to more rapid induction of the reporter mRNA, export of the message to the cytoplasm, and the subsequent accumulation of the encoded reporter protein, which also can affect transcriptional and post-transciptional processes^84^. Thus, we argue that the post-transcriptional regulation resulted in the difference between ambient CO_2_ and elevated CO_2_ treatments within a given N level.

Several explanations have been given that DNA methylation plays an important role in regulating gene expression in transgenic plants as well as plants in general^34,35,85^. Weinhold *et al*.^34^ noted that three independently transformed tobacco lines rapidly lost the expression of the resistance marker and down-regulated transgene expression by more than 200 fold after only one plant generation, while at the same time, the hypermethylation status was displayed within the 35 S and NOS promoters of these lines. Additionally, different methylation patterns (CG, CHG and CHH) may also play an important role in regulating gene expression^34,35,86^. Previous studies have also indicated that the methylation status may be influenced by external factors, including virus infection^35^ or nutrition-induced^87^ plant response factors. Furthermore, environmental factors are also considered as potential inducers to change the methylation status. For example, a sudden cold environment has been shown to trigger the demethylation of hemi-methylated or internally full methylated cytosine in cotton, and this change could be reversed following a subsequent normal temperature^88^. However, unlike the promoter region, loss of body methylation does not appear to trigger a systematic and drastic over-expression of body-methylated genes to the same extent as transposon reactivation, whereas moderate regulation of body-methylated genes has been observed, suggesting that body methylation may be involved in fine-tuning transcription levels^89,90^. Our results seem to concur with this phenomenon, which may show a moderate regulation to the expression of *Bt* gene, because the methylation remain at relatively low level in this study.

In summary, transgene expression in plants is characteristically unpredictable, and depends on many internal and external factors. In this study, the expression of *Bt* protein toxin transferred in rice was relatively stable under elevated CO_2_ at different N levels, except at 2 N level. It means there are no significant effects on exogenous *Bt* toxin affected by elevated CO_2_ under same N level (below standard N level), but under high N level, elevated CO_2_ shows promotive effect. In the foreseeable future, it is not expected that the global atmospheric CO_2_ concentrations will trigger a significant down-regulation of transgene expression in transgenic *Bt* rice and appropriate increase of N fertilization may be prudent for its expression. It is noteworthy that the foreign protein expression is not reduced under N limitation production scenarios, although the plant growth is restricted and total protein content of the plant is reduced. Ruiz *et al*.^65^ showed that the subjecting of genetically modified plants to induction conditions drives the production and accumulation of the recombinant phytotoxic molecule, which in turn progressively damages host cells. This damage depends on the concentration and the length of exposure to the newly synthesized molecule, consequently negatively impacting the biomass and yield. With the development of genetic engineering, the mechanism of exogenous gene expression and post-transcriptional regulation will be further studied. And it is expected that N fertilization supply may promote the expression of *Bt* toxin in transgenic Bt rice, particularly under elevated CO_2_.

## Electronic supplementary material

Supplementary Information
