# Supplementary material for: Impacts of elevated CO2 on exogenous Bacillus thuringiensis toxins and transgene expression in transgenic rice under different levels of nitrogen
Source: Sci Rep. 2017 Nov 7;7:14716. doi: 10.1038/s41598-017-15321-9 (PMC5676734; doi:10.1038/s41598-017-15321-9)
Supplement: Supplementary file 1 — Supplementary Information [file 41598_2017_15321_MOESM1_ESM.pdf]

Impacts of elevated CO_2 on exogenous *Bacillus thuringiensis* toxins and transgene expression in transgenic rice under different levels of nitrogen

Shoulin Jiang[1], Yongqing Lu[1], Yang Dai[1], Lei Qian[1], Adnan Bodlah Muhammad[1], Teng Li[1], Guijun Wan[1], Megha N. Parajulee[2], Fajun Chen[1*]

[1]Department of Entomology, Nanjing Agricultural University, Nanjing 210095, China;
[2]Texas A&M University AgriLife Research and Extension Center, Lubbock, TX, USA

Supplementary legends:

Supplementary Fig. 1 Methylation maps of P1 fragment in the fused *Cry 1Ab/Ac* transgene region in leaves of transgenic *Bt* rice grown under ambient CO_2 with different N-fertilizer rates. (n=minimum of 10 clones for each treatment. Positions of cytosines (CGN, CHG and CHH) on the analyzed regions are represented in Fig. 7. Filled symbols indicate cytosine methylation. Red, blue and green circles represent CGN, CHG and CHH sites respectively, whereas H = A, T or C.

Supplementary Fig. 2 Methylation maps of P1 fragment in the fused *Cry 1Ab/Ac* transgene region in leaves of transgenic *Bt* rice grown under elevated CO_2 with different N-fertilizer rates. (n=minimum of 10 clones for each treatment. Positions of cytosines (CGN, CHG and CHH) on the analyzed regions are represented in Fig. 7. Filled symbols indicate cytosine methylation. Red, blue and green circles represent CGN, CHG and CHH sites respectively, whereas H = A, T or C.

Supplementary Fig. 3 Methylation maps of P2 fragment in the fused *Cry 1Ab/Ac* transgene region in leaves of transgenic *Bt* rice grown under ambient CO_2 with different N-fertilizer rates. (n=minimum of 10 clones for each treatment. Positions of cytosines (CGN, CHG and CHH) on the analyzed regions are represented in Fig. 7. Filled symbols indicate cytosine methylation. Red, blue and green circles represent CGN, CHG and CHH sites respectively, whereas H = A, T or C.

Supplementary Fig. 4 Methylation maps of P2 fragment in the fused *Cry 1Ab/Ac* transgene region in leaves of transgenic *Bt* rice grown under elevated CO_2 with different N-fertilizer supply (n=minimum of 10 clones for each treatment. Positions of cytosines (CGN, CHG and CHH) on the analyzed regions are represented above. Filled symbols indicate cytosine methylation. Red, blue and green circles represent CGN, CHG and CHH sites respectively, whereas H = A, T or C.

* Correspondence: Fajun Chen, Department of Entomology, Nanjing; Agricultural University, Nanjing 210095, China. E-mail: fajunchen@njau.edu.cn

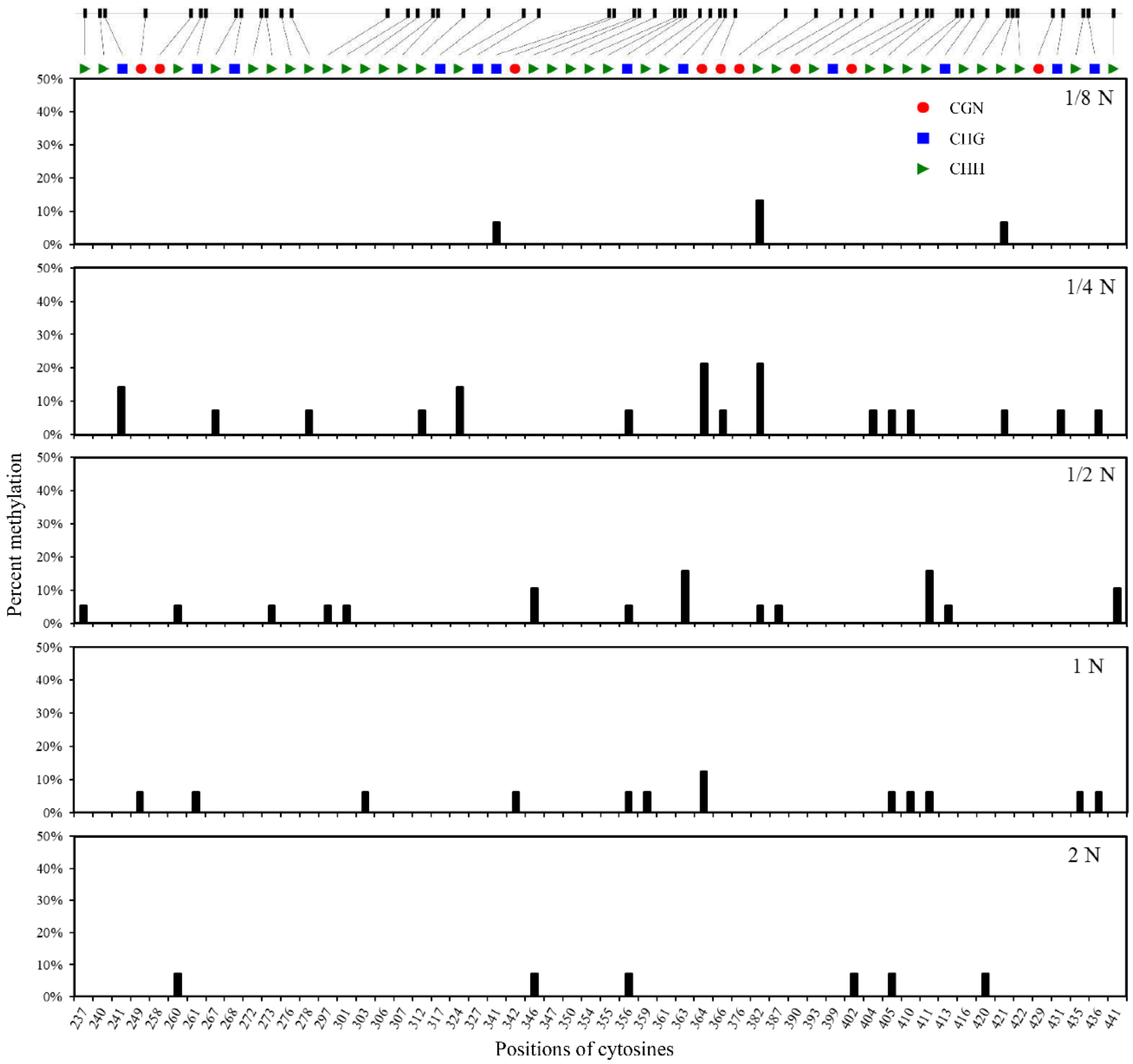

Supplementary Fig. 1 Methylation maps of P1 fragment in the fused *Cry 1Ab/Ac* transgene region in leaves of transgenic *Bt* rice grown under ambient CO_2 with different N-fertilizer rates (n=minimum of 10 clones for each treatment. Positions of cytosines (CGN, CHG and CHH) on the analyzed regions are represented in Fig. 7. Filled symbols indicate cytosine methylation. Red, blue and green circles represent CGN, CHG and CHH sites, respectively, whereas H = A, T or C).

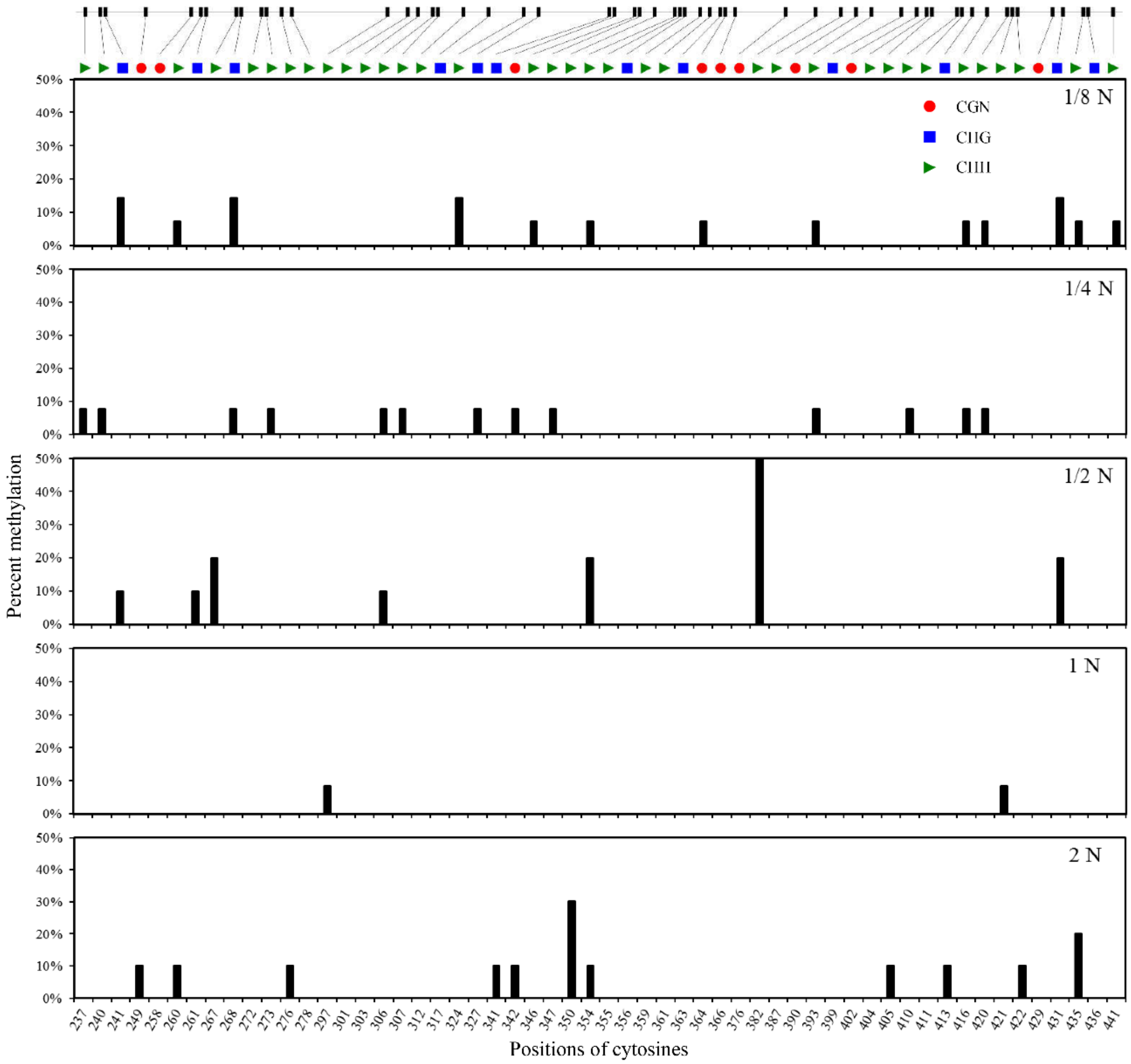

Supplementary Fig. 2 Methylation maps of P1 fragment in the fused *Cry 1Ab/Ac* transgene region in leaves of transgenic *Bt* rice grown under elevated CO_2 with different N-fertilizer rates (n=minimum of 10 clones for each treatment. Positions of cytosines (CGN, CHG and CHH) on the analyzed regions are represented in Fig 7. Filled symbols indicate cytosine methylation. Red, blue and green circles represent CGN, CHG and CHH sites respectively, whereas H = A, T or C).

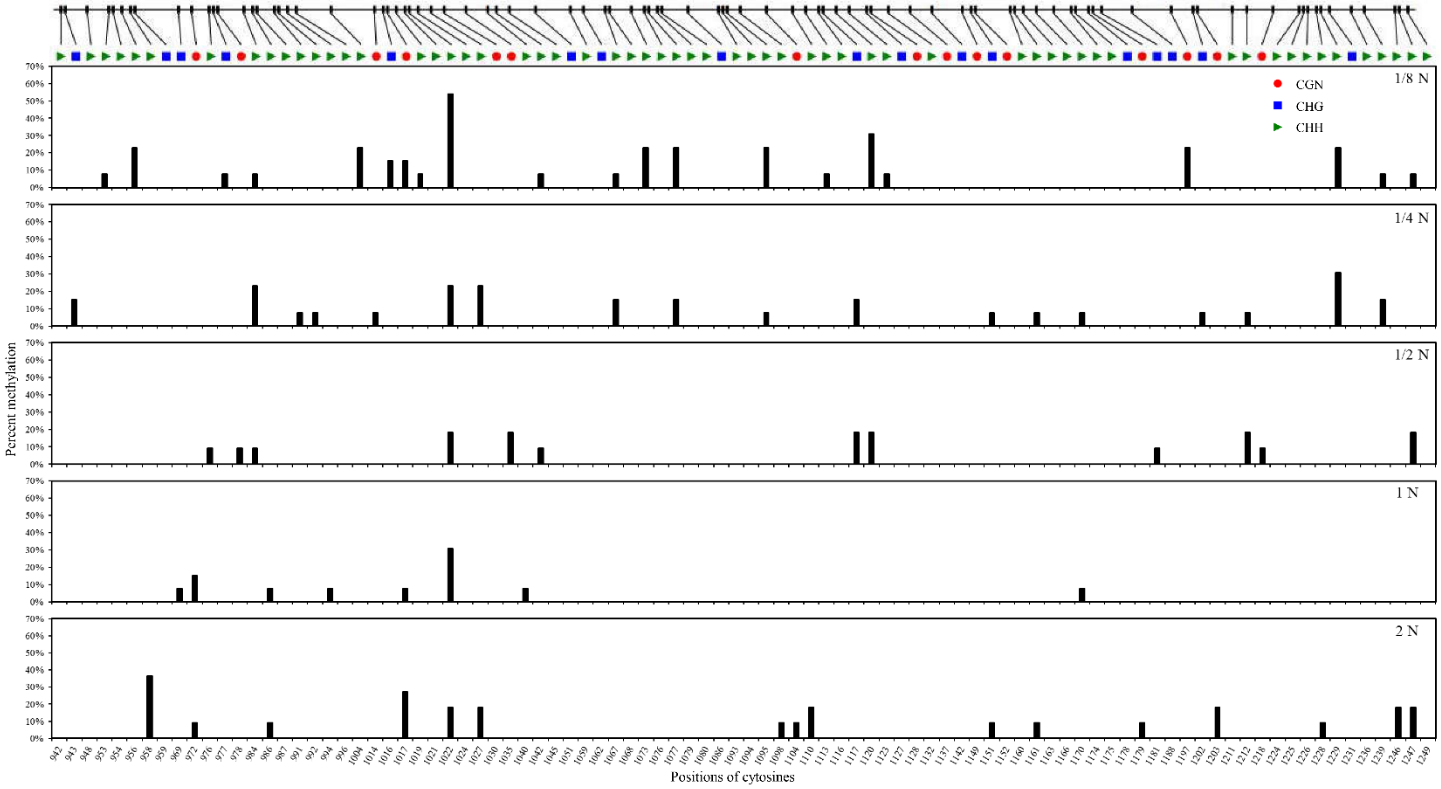

Supplementary Fig. 3 Methylation maps of P2 fragment in the fused *Cry 1Ab/Ac* transgene region in leaves of transgenic *Bt* rice plant grown under ambient CO_2 with different N-fertilizer supply (On the average of at least 10 clones for each treatment. Positions of cytosines (CGN, CHG and CHH) on the analyzed regions are represented above. Filled symbols indicate cytosine methylation. Red, blue and green circles represent CGN, CHG and CHH sites respectively, whereas H = A, T or C.)

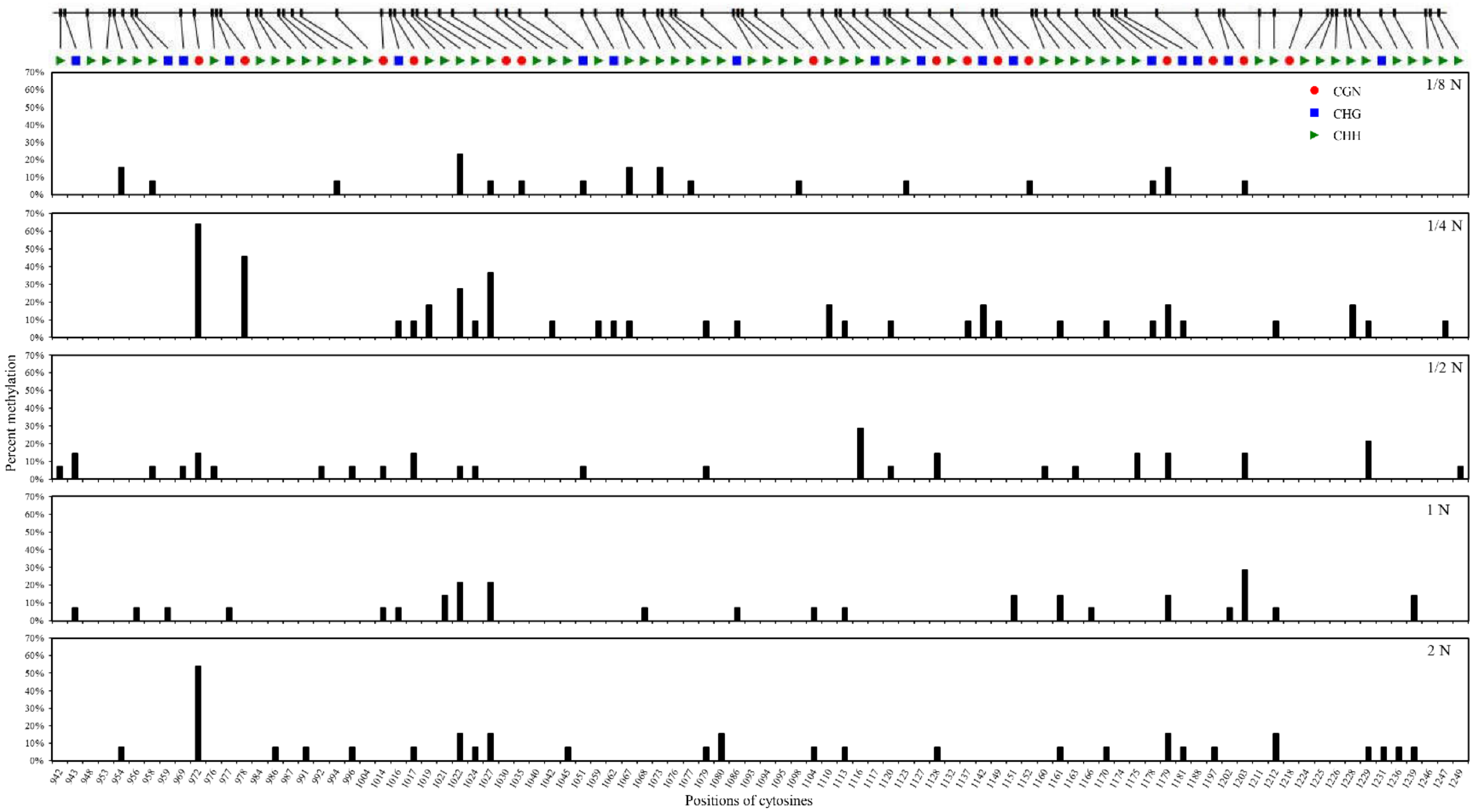

Supplementary Fig. 4 Methylation maps of P2 fragment in the fused *Cry 1Ab/Ac* transgene region in leaves of transgenic *Bt* rice plant grown under elevated CO_2 with different N-fertilizer supply (On the average of at least 10 clones for each treatment. Positions of cytosines (CGN, CHG and CHH) on the analyzed regions are represented above. Filled symbols indicate cytosine methylation. Red, blue and green circles represent CGN, CHG and CHH sites respectively, whereas H = A, T or C.)
